# Patterns of Adherence to and Compliance with the Portuguese Smoke-Free Law in the Leisure-Hospitality Sector

**DOI:** 10.1371/journal.pone.0102421

**Published:** 2014-07-18

**Authors:** Maria Fátima Reis, Sónia Namorado, Pedro Aguiar, José Precioso, Baltazar Nunes, Luís Veloso, Sandra Santos, José Pereira Miguel

**Affiliations:** 1 Instituto de Saúde Ambiental, Faculdade de Medicina da Universidade de Lisboa, Lisboa, Portugal; 2 Instituto de Medicina Preventiva, Faculdade de Medicina da Universidade de Lisboa, Lisboa, Portugal; 3 Escola Nacional de Saúde Pública, Universidade Nova de Lisboa, Lisboa, Portugal; 4 Instituto de Educação, Universidade do Minho, Braga, Portugal; 5 Departamento de Epidemiologia, Instituto Nacional de Saúde Doutor Ricardo Jorge, Lisboa, Portugal; 6 Clinical Data Unit, Eurotrials, Lisboa, Portugal; Newcastle University, United Kingdom

## Abstract

**Background:**

In 2008, the Portuguese smoke-free law came into effect including partial bans in the leisure-hospitality (LH) sector. The objective of the study is to assess the prevalence of smoking control policies (total ban, smoking permission and designated smoking areas) adopted by the LH sector in Portugal. The levels of noncompliance with each policy are investigated as well as the main factors associated with smoking permission and noncompliance with the law.

**Methods:**

Cross-sectional study conducted between January 2010 and May 2011. A random sample of venues was selected from the Portuguese LH sector database, proportionally stratified according to type, size and geographical area. All venues were assessed *in loco* by an observer. The independent effects of venues' characteristics on smoking permission and the level of noncompliance with the law were explored using logistic regression.

**Results:**

Overall, 1.412 venues were included. Total ban policy was adopted by 75.9% of venues, while 8.4% had designated smoking areas. Smoking ban was more prevalent in restaurants (85.9%). Only 29.7% of discos/bars/pubs opted for complete ban. Full or partial smoking permission was higher in discos/bar/pubs (OR = 7.37; 95%CI 4.87 to 11.17). Noncompliance with the law was higher in venues allowing smoking and lower in places with complete ban (33.6% and 7.6% respectively, p<0.001). Discos/bars/pubs with full smoking permission had the highest level of noncompliance (OR = 3.31; 95%CI 1.40 to 7.83).

**Conclusions:**

Our findings show a high adherence to smoking ban policy by the Portuguese LH sector. Nonetheless, one quarter of the venues is fully or partially permissive towards smoking, with the discos/bars/pubs considerably contributing to this situation. Venues with smoking permission policies were less compliant with the legislation. The implementation of a comprehensive smoke-free law, without any exceptions, is essential to effectively protect people from the second hand smoke.

## Introduction

The accumulated evidence suggests that exposure to second-hand smoke (SHS) contributes to a range of serious and fatal conditions including lung cancer, cardiovascular diseases and respiratory symptoms. Moreover, it is firmly established that there is no safe level of exposure.[1], [2] Of all public places, restaurants, bars and discos have the highest levels of exposure to SHS, posing their workers to serious health risks.[3]–[5] As a consequence, governments worldwide are increasingly implementing smoke-free laws in all enclosed public places and workplaces to protect people, including workers, from the harmful effects of SHS exposure.[2], [6] Previous research showed that total bans are the only effective way to reduce exposure and to effectively protect the population.[7], [8] This has been achieved in several countries through well-planned education campaigns that reinforced public support and comprehensive active enforcement of the law.[9]–[11]

The Portuguese smoke-free law (law 37/2007) came into force on January 2008 targeting all indoor public places and workplaces.[12] However, in the leisure-hospitality (LH) sector this law has not been totally effective in creating smoke-free environments due to a partial ban, with ambiguities and exemptions, following in part the old “Spanish model".[13], [14] Public venues smaller than 100 m^2^ can allow smoking, provided ventilation and exhaust systems are in place, while venues larger than 100 m^2^ are compulsorily smoke-free but can adopt designated smoking areas (DSA) if these do not exceed 30% of the total area. Besides, DSA should be physically separated from the non-smoking areas or have individual ventilation systems. In either case, the removal of exhaust air has to be ensured.[12]

Evidence shows that either partial or full smoking permission policies are ineffective in both protecting the health of workers and preventing non-smokers' exposure to SHS.[8], [15]–[17] Furthermore, the smoking areas may intensify smokers' exposure and fail to contribute either in reducing smoking initiation or in facilitating smokers to cut down or quit.[8], [18]

Few studies have concurrently investigated the pattern of adherence and the level of compliance with legislation that includes partial smoking restrictions, especially in countries with poor tobacco control policies such as Portugal.[19]

After three years of experience with the 2007 smoke-free legislation it was considered essential to evaluate the adherence of the LH sector to each of the smoking control policies (total ban, full permission, DSA) and the level of compliance with the law.

The aim of our study was to assess the prevalence of the smoking control policies of the Portuguese legislation adopted by the national LH sector. In addition, we also investigated the levels of noncompliance to each policy as well as the main factors associated with the noncompliance.

## Methods

### Ethics statement

The study was approved by the Ethics Committee of Faculdade de Medicina de Lisboa.

### Study design

We conducted an observational cross-sectional study between January 2010 and May 2011 using a planned representative sample of venues from the LH sector in Portugal. The data was mostly collected between March and June 2010 (for 91% of the venues). A two-step approach was used. First, a proportional stratified sample of LH venues was randomly selected. Second, an observation *in loco* was conducted to collect information of interest from each venue.

### Sample

The sample for the present study was drawn from the Ministry of Labour and Social Solidarity database (2008), where the venues are identified by locality, postal code, type (restaurants, cafeterias/pastries and discos/bars/pubs) and number of workers. The later was used as a preliminary estimate of venue size (number of workers ≥10 = 100 m^2^ or over) which was confirmed during the observation *in loco*. Locality was defined as the geographic area that includes the county and all its civil parishes (urban or rural) of each of the 28 NUTS-3 (Nomenclature of Units for Territorial Statistics - Level 3), which are subregions of NUTS-2 regions of Continental Portugal (North, Centre, Lisbon, Alentejo and Algarve). The sample included the most populated localities from each NUTS-3 as well as the district capitals when these did not coincide with the NUTS-3 most populated localities.[20] Overall, 32 localities were included, proportionally stratified by NUTS-2 regions. For each locality, 40 venues were randomly selected based on a proportional quota sampling according to the venue's type and size. This target number was defined to ensure a minimum of 30 sampling units per locality, considering eventual sample loss. A sample of 1492 venues was identified, allowing the estimation of a general proportion of adherence with a confidence interval of 95% and a margin of error of 2.5%.

The planned distribution of venues according to type, size and region (NUTS-2) was the following: cafeteria/pastries (38%), restaurants (54%) and discos/bars/pubs (8%); venues <100 m^2^ [small venues] (89%); North (32%), Centre (20%), Lisbon (35%), Alentejo (6%) and Algarve (7%). In our sample, we observed the following distribution: cafeteria/pastries (40%), restaurants (49%) and discos/bars/pubs (11%); small venues (89%); North (28%), Centre (35%), Lisbon (15%), Alentejo (16%) and Algarve (6%). This sample showed a good representativeness regarding type and size. Regarding the NUTS-2, although the proportional distribution was not entirely met, all of the five Portuguese regions were represented in our sample.

### Observation *in loco* and data collection

All selected venues were assessed *in loco* once by an observer for a complete outdoor and indoor characterisation. When the type or size of the venue observed had no correspondence with pre-existing data, another venue was sequentially selected until the quota for that locality was fulfilled. The same observer was assigned to each locality.

The observations were performed during the periods of maximum public attendance, taking in to account the type of setting (e.g. discos/bars/pubs were observed late-night Friday or Saturday only) and took at least five minutes (maximum duration depended if the observer recorded the data from outdoors or indoors). During the assessment the observers made all the efforts to make their presence unknown to the owners/workers of venues.

All observers (25 in total) had an academic degree and were highly familiar with the locality. They were appropriately trained by the same researcher through an individual phone session, during which study procedures were explained and a vast set of case-scenarios were discussed. Each observer was provided with a unique, detailed study protocol (mainly based in flowcharts) and followed a standardized questionnaire designed to collect the following information: type and size of the venue (small <100 m^2^; large ≥100 m^2^), maximum occupancy, smoking control policy adopted (assessed through the presence of non-smoking/smoking signage or the existence of DSA). The observers had to report the status of the fieldwork on a daily basis and were instructed to raise any queries to the researchers, whenever necessary.

Relevant indicators of noncompliance with smoke free-law requirements were collected, tailored to the smoking control policy adopted by each venue. These included the existence/visibility and adequacy of the prescribed signage, the existence of ventilation/exhaust systems and their operating status (on/off) and the visual and olfactory evidence of tobacco use (Table 1). These indicators allowed the determination of a level of noncompliance with the law, taking into account the smoking policy adopted. Each indicator was rated by the observer contributing to an overall score of noncompliance for each venue.

**Table 1:** Indicators of noncompliance with the Portuguese smoke-free law according to the smoking control policy.

**Smoking ban** (score range: 0–18)
a. Inexistence/no visibility of the signage (red display) from outdoors
b. Characteristics related to the conformity of the signage not verified (3 sub-items):
b.1. Dimension (≥160 mm ×55 mm)
b.2. Label with reference to the smoke-free law
b.3. Reference to the penalty for violating the smoking prohibition
c. People smoking
d. Ashtray
e. Tobacco smell
f. Cigarette butts
**Designated smoking areas** (score range: 0–24)
a. Inexistence/no visibility of the signage (blue and red displays) from outdoors
b. Characteristics related to the conformity of the signage not verified (2 sub-items):
b.1. Dimension
b.2. Label with reference to the smoke-free law
c. No specific identification in smoking and non-smoking areas
d. No physical separation between smoking and non-smoking areas
e. People smoking in non-smoking areas
f. Environmental smoke in non-smoking areas
g. Tobacco smell in non-smoking areas
h. Other evidence of smoking in non-smoking areas
**Full smoking permission** (score range: 0–9)
a. Inexistence/no visibility of the signage (blue display) from outdoors
b. Characteristics related to the conformity of the signage not verified (2 sub-items):
b.1. Dimension (≥160 mm ×55 mm)
b.2. Label with reference to the smoke-free law
c. Inexistence/off status of the ventilation system

Each indicator of noncompliance was rated as 0 or 3 (compliance or noncompliance) and each sub-item was scored 0 (compliant) or 1 or 1.5 (non-compliant, for three or two sub-items, respectively).

Since distinct indicators were used for each smoking policy it was necessary to use a relative measure of noncompliance in order to make policies comparable with each other regarding this variable. The rate of noncompliance was defined as the ratio of the overall score attributed to a venue over the maximum score possible, according to the policy adopted (0% and 100% corresponding to the lowest and to the highest rate of noncompliance, respectively).

We also explored the effect of the type, size and region of the venues on the presence of relevant noncompliance according to the smoking policy adopted. For this purpose, a relevant level of noncompliance was considered for rates above the sample mean (RAM). RAM = 1 was defined when the rate of noncompliance attributed to a venue with a particular policy was greater than or equal to the corresponding group mean; otherwise RAM = 0 was considered.

### Statistical analysis

Descriptive statistics and frequency tables were used for the characterization of venues and smoking policy adopted. Adherence was assessed through the frequency that venues adopted each of the smoking policies provided in the Portuguese smoke free-law. The results of venues with smoking permission were aggregated with those with DSA, both representing smoking permission ( = 1), and compared with venues with total ban policy ( = 0). Chi-squared or Kruskal-Wallis tests were used for comparisons between categorical and numerical variables, respectively. Odds Ratio (OR; 95%CI) were used to measure associations between venue's characteristics (type, size and region) and the following dependent variables: smoking control policy (total ban or full permission + DSA) and relevant level of noncompliance (RAM). Breslow-Day test was used to assess the homogeneity of OR across strata and to detect interaction effect between venue's type and size.

Adjusted OR were assessed by multivariable logistic regression models to analyse the association between venue's characteristics and dependent variables of interest: smoking control policy and noncompliance RAM. To be included in the regression models, the variables had to cumulatively meet the following criteria: no evidence of colinearity; p<0.10 in the bivariable analysis; and a rate of missing values <10%. The models were optimized through backwards methods and goodness-of-fit was assessed using the Hosmer and Lemeshow test and the area under the Receiver Operating Characteristics (ROC) curve.

Statistical significance was set at 5%. *IBM SPSS* for *Windows* version 20 was used for all analyses.

## Results

### Adherence to smoking control policies

The adopted smoking control policy was reported for 1.394 venues (Table 2). In 18 venues the observer could not draw a conclusion about the type of smoking control policy adopted. The majority of the venues (75.9%) adopted a total ban policy, while 15.7% opted for smoking permission and 8.4% had DSA. The adoption of total ban policy was more frequent in restaurants (85.9%), and less frequent in discos/bars/pubs (29.7%). DSA were found in 21.3% of large venues. The full permission policy was more prevalent in small venues (16.5%) when compared to large ones (9.3%). The prevalence of the total ban policy was higher in Lisbon region (86.3%) while Alentejo adopted full permission policy more frequently (18.8%). DSA were more common in the Centre region (12.2%).

**Table 2:** Prevalence of smoking policies by type, size and region of the venue, and OR derived from bivariable and multiple logistic regression.

			Smoking control policy					
		(n)	Total ban (%)	Smoking permission (%)	DSA (%)	Smoking permission + DSA (%)	Crude OR (95%CI)	Adjusted OR (95%CI)
	Total of venues	1394^a^	75.9%	15.7%	8.4%	24.1%		
Type of venue	Cafeteria/pastry	563	75.7%	19.0%	5.3%	24.3%	Reference	Reference
	Restaurant	686	85.9%	5.8%	8.3%	14.1%	0.51 (0.38–0.68)^b^	0.48 (0.36–0.64)^b^
	Disco/bar/pub	145	29.7%	49.7%	20.7%	70.3%	7.38 (4.92–11.06)^b^	7.37 (4.87–11.17)^b^
Size	Small (<100 m^2^)	1244	76.7%	16.5%	6.8%	23.3%	Reference	Reference
	Large (≥100 m^2^)	150	69.3%	9.3%	21.3%	30.7%	1.46 (1.11–1.91)^b^	1.66 (1.09–2.54)^b^
NUTS-2 region	North	427	80.6%	14.3%	5.2%	19.4%	Reference	Reference
	Centre	525	69.9%	17.9%	12.2%	30.1%	1.78 (1.32–2.42)^b^	1.96 (1.41–2.73)^b^
	Lisbon	131	86.3%	9.2%	4.6%	13.7%	0.66 (0.38–1.15)	0.65 (0.36–1.18)
	Alentejo	223	71.3%	18.8%	9.9%	28.7%	1.67 (1.15–2.43)^b^	1.83 (1.22–2.76)^b^
	Algarve	88	85.2%	11.4%	3.4%	14.8%	0.72 (0.38–1.36)	0.65 (0.32–1.33)

a Sample losses were due to places that had either closed or changed type by the time of observation *in loco* and no similar unit was available in the database.

b p<0.001.

Chi-square test was used for comparison of proportions of venues characteristics (type, size and regions) for each smoking policy.

95%CI: 95% Confidence interval.

Model p-value (p<0.001); Hosmer and Lemeshow test (p = 0.066); Area under the ROC curve  = 73.7% (95% CI: 70.6%–76.9%).

DSA – Designated Smoking Area.

### Comparative analysis between venues' characteristics regarding smoking control policy

With the exception of Lisbon and Algarve regions, all variables related to the venue's characteristics showed statistically significance in the bivariable analysis (Table 2). Full or partial smoking permission was higher in discos/bar/pubs (OR = 7.37), large venues (OR = 1.66), and in Centre (OR = 1.96) and Alentejo (OR = 1.83) regions, when compared with the references cafeteria/pastry, smaller venues and the North region, respectively. Restaurants were less permissive towards smoking than cafeterias/pastries (OR = 0.48).

Multivariable adjusted ORs for type, size and region of the venue were highly consistent with the results obtained in the bivariable analysis.

### Evaluation of noncompliance with the smoke-free law

The proportions found for each indicator of noncompliance are presented as Supporting Information (Table S1). Of note, the signage was not visible from outdoors in approximately 15% of the venues observed, independently of the smoking policy adopted. Furthermore, outdoor visibility of the signage was more frequent in venues that adopted a total ban policy.

Table 3 shows that the smoking control policies are highly associated with the rate of noncompliance with the smoke-free law requirements. Noncompliance was higher in the venues where smoking was fully permitted (33.6%) and was lower in those venues that adopted a total ban policy (7.6%).

**Table 3:** Rates of noncompliance according to smoking control policy.

	Rate of noncompliance^a^					
Smoking control policy	n	Mean (95%CI)	Median	SD	Min-Max	p-value^b^
Total ban	1058	7.6 (6.7–8.9)	0.0	16.2	0–100	<0.001
Smoking permission	219	33.6 (30.0–37.2)	33.3	27.1	0–100	
DSA	117	19.7 (16.1–23.3)	12.5	19.7	0–87.5	

a The rate of noncompliance was defined as the ratio of the overall score attributed to a venue over the maximum score possible, according to the smoking policy adopted (0% =  lowest rate of noncompliance; 100% =  highest rate of noncompliance).

b Kruskall-Wallis Test.

DSA – Designated Smoking Area; SD – Standard Deviation.

The bivariable analysis showed a statistically significant association between the noncompliance RAM and the type of venue (Table 4). Discos/bars/pubs showed the highest noncompliance RAM which was more notable in those venues with DSA (56.7%). Conversely, restaurants had the lowest noncompliance RAM, when total ban was adopted (17.8%). No statistical significant association was found between the venue's size and noncompliance RAM. The association between venue's region and noncompliance RAM was statistically significant only when the smoking ban option was adopted (p<0.001). Among this subgroup, the venues located in Alentejo showed the highest noncompliance RAM (37.1%).

**Table 4:** Logistic regression predicting the RAM adjusted by type, size and NUTS-2 regions for each smoking control policy.

		Total ban		Full smoking permission		DSA	
Independent variable		Noncompliance ≥ Mean (RAM)	Adjusted OR (95%CI)	Noncompliance ≥ Mean (RAM)	Adjusted OR (95%CI)	Noncompliance ≥ Mean (RAM)	Adjusted OR (95%CI)
**Type of venue**	Cafeteria/Pastry	25.4%	Reference	9.3%	Reference	46.7%	Reference
	Restaurant	17.8%	0.637 (0.467–0.871) c	15.0%	1.569 (0.517–4.762)	28.1%	0.266 (0.093–0.767)^c^
	Disco/Bar/Pub	32.6%	1.316 (0.659–2.627)	26.4%	3.308 (1.398–7.825)^c^	56.7%	1.191 (0.397–3.571)
	p-value^a^	0.003		0.009		0.025	
**Size of venue**	Small (<100 m^2^)	22.1%	Reference	21.4%	Reference	53.1%	Reference
	Large (≥100 m^2^)	21.4%	1.164 (0.704–1.924)	15.6%	1.086 (0.267–4.415)	35.3%	2.304 (0.903–5.881)
	p-value^a^	0.904		0.565		0.079	
**NUTS-2 Region**	North	17.4%	Reference	19.7%	Reference	31.8%	Reference
	Centre	18.0%	1.082 (0.733–1.598)	10.6%	0.550 (0.217–1.395)	34.4%	1.245 (0.412–3.766)
	Lisbon	19.5%	1.162 (0.674–2.006)	16.7%	0.749 (0.139–4.046)	50.0%	2.096 (0.295–14.903)
	Alentejo	37.1%	2.786 (1.814–4.278)^c^	19.0%	1.152 (0.405–3.277)	60.0%	4.881 (1.292–18.438)^b^
	Algarve	26.7%	1.812 (1.002–3.277) c	30.0%	1.644 (0.352–7.675)	---	---
	p-value^a^	<0.001		0.357		0.118	
	Model p-value		<0.05		<0.05		<0.05
	Hosmer and Lemeshow Test		0.797		0.774		0.572
	Area under the ROC curve		62.4%		67.5%		72.7%
	(95%CI)		(58.3%–66.6%)		(57.4%–77.7%)		(63.3%–82.1%)

a Chi-squared test for comparison of levels of noncompliance with the law between venues characteristics (type, size and region) for each smoking policy.

b Alentejo and Algarve were grouped for venues with DSA because Alentejo presented cells with zero expected count.

c p<0.05.

95%CI: 95% Confidence interval.

DSA – Designated Smoking Areas.

RAM – Rate above the mean.

The multivariable analysis showed that the strength of association between some of the statistically significant predictors of the bivariable analysis and the noncompliance RAM varied according to the different policies adopted. Restaurants were more compliant when total ban and DSA policies were adopted (OR = 0.64 and OR = 0.25, respectively; p<0.001). The association between discos/bar/pubs and the noncompliance RAM was not statistically significant, except when full smoking permission was adopted (OR = 3.31; p<0.001).

Whichever the policy adopted the adjusted association between venue's size and noncompliance RAM remained non-statistically significant. Adjusted association between venue's region and noncompliance RAM was statistically significant in venues in Alentejo (OR = 2.79; p<0.001) and Algarve (OR = 1.81; p<0.001) that adopted total ban policy and in venues in Alentejo with DSA (OR = 4.89; p<0.001).

The regression analysis to investigate the interaction between venue's size and type regarding the noncompliance RAM showed no effect modification for any of the smoking control policies.

## Discussion

This study is the first to investigate the adherence of LH sector to the 2007 Portuguese smoke-free law on a nationwide scale, three years after it came into force.

Our findings show that the vast majority of venues from this sector (76%) adopted a total ban policy, independently of the type, size or region. This represents a high level of adherence to a more restrictive legislation from the owners of LH venues and is consistent with previous research in Portugal. Two exploratory regional studies conducted within the first year after the law came into effect, showed that adherence to smoking ban varied between 71% and 77%.[21], [22] Furthermore, opinion polls revealed not only a high support to the smoking ban from the Portuguese population but also their view that the law was being complied.[14] One cohort study conducted in Spain to explore the expectations and attitudes of LH workers towards the smoking legislation, before and two years after it came into force, revealed an increased support to the ban policy in all public places including bars and restaurants from 54.1% to 65.8%.[23] Nonetheless, the proportions observed in our study are considerably lower than the ones reported in countries such as Ireland. This country was the first (2004) to implement a nationwide smoking ban in all workplaces, including bars and restaurants. Since then, the Irish case constitutes a paradigm of how political measures such as smoking legislation can positively affect the public health. A report from the Irish Office of Tobacco Control, one year after the legislation came into force, suggested that 96% of the population surveyed (including 89% of smokers) felt that the smoking law was successful. In addition, support was more striking in venues where pre-policy support was lowest (bars and restaurants).[24] This social support, in a country where drinking and smoking is part of the cultural tradition, shows that the majority of people in spite of their lifestyles yearn for healthy environments in their work and recreational places.

Noticeably, more than a quarter of venues from the LH sector in Portugal were fully or partially permissive towards smoking, with the highest prevalence found in the Centre and Alentejo regions. This means that a considerable segment of owners/workers and costumers in these settings are potentially exposed to SHS and to its harmful effects. From a public health perspective this finding highlights the importance to promote awareness campaigns, particularly in these two regions of Portugal, in order to better protect people from SHS exposure.

The Spanish and the Uruguay experience are elucidatory. In Spain the tobacco law implemented in 2006 banned smoking in all workplaces except for LH venues, where partial restrictions were applied depending on the size of the venue.[25] Several follow up studies found that workers and customers from LH sector were still exposed to extremely high levels of tobacco smoke two years after the implementation of the smoking legislation.[17], [26]–[28] This led Spanish legislators to change the smoke-free law in January 2011, extending the total ban to all enclosed areas including bars, discos, restaurants and even hospitals campus and healthcare centers. In 2012, Lopez et al. showed that SHS exposure in LH settings was dramatically reduced after the 2011 Spanish smoking ban was introduced.[29] High rates of compliance with smoke-free law (92.3%) were also observed in public places of a North India district, revealing the effectiveness of ban policies.[11] In 2006, Uruguay was the first Latin American country to implement a nationwide smoke-free policy, as result of successful experiences worldwide with smoking ban legislations, coupled with a strong political support from the President of the Republic.[30] In a recent investigation, Sureda et al. (2013) observed that the benefits of smoking ban in reducing SHS exposure also extended to other settings such as home, contradicting the speculative hypothesis from the tobacco industry of displacement of smoking from public to private places. [31]

Of note, approximately 7% of small venues (<100 m^2^) observed had smoking areas. This could be attributed to owners' lack of awareness of the smoke-free legislation or a mis-estimation of venues size.

Our study also revealed that the rates of adoption of each smoking policy vary according to the type of venue. Restaurant is the setting with the highest adherence to the total ban policy while discos/bars/pubs are the most permissive towards smoking. This is not surprising since several country-level and multi-country surveys of public attitudes towards smoke-free policies have shown that support for smoke-free restaurants is consistently higher than for bars, particularly among smokers.[32] Nevertheless, experience from some countries demonstrates a marked increase of support for total bans in bars after its implementation. A survey in California found that bar owners and staff became increasingly supportive of smoke-free environment after restrictions were introduced.[33] Similar trends were observed in New Zealand with the support increasing from 44% to 60%.[34]

An overview of the indicators of noncompliance showed us that venues, in general, are highly compliant with the signage visibility from outdoors but especially those venues that adopted a smoking ban policy. This is of importance as this signage consists of the first information about the smoking policy adopted by a particular venue that reaches the costumers. Conversely, places with DSA are the less compliant with this aspect.

The evaluation of compliance with smoke-free legislation is an important objective indicator of its social acceptance. Our results show that the level of venues' compliance with the law is highly dependent on the smoking control policy adopted. Venues with smoking permission policies (full permission or DSA) showed higher levels of noncompliance than venues that adopted ban policy. Overall, these results are in line with previous research conducted in the Portuguese LH sector.[35]

It was also observed that the type of venue was statistically associated with noncompliance RAM. Discos/bars/pubs had the highest rates of noncompliance, which was more marked in places with DSA (RAM = 56.7%), while restaurants were the most compliant venues, except when no smoking restrictions were in place. In addition, restaurants were more compliant when total ban and DSA policies were adopted.

Scientific evidence shows that partial bans are difficult to comply while comprehensive legislations are easier to implement and enforce.[36], [37] In Chile, a large segment of the LH sector that adopted partial smoking restrictions experienced difficulties in ensuring customer compliance.[38] In Spain, the level of compliance of the LH sector with the partially restrictive law of 2006 was controversial and highly variable across regions, in part explained by several exceptions that led to misinterpretations and difficulties in assessing compliance.[39]

Conversely, in Ireland, a country with more restrictive smoking control policies, inspections and sanctions data, one year after the law came into effect, revealed high levels of compliance, ranging from 89% in pubs to 98% in restaurants.[24]

Compliance with the smoke-free legislation is critical to its effectiveness in protecting people from exposure to SHS. To achieve high levels of compliance it is crucial that governments ensure an active and uniform enforcement, at least until the legislation becomes self-enforcing^1^. This is particularly important in countries with legislation that includes exceptions and leaves room for interpretations and is the case of Portugal.[22]

### Strengths and limitations

One of the major strengths of our research was the planned random sample of venues, proportionally stratified by type, size and region, which was expected to confer a high level of representativeness of the LH sector in continental Portugal. The observed sample was proportionally represented in respect to size and type. Although the venues' NUTS-2 distribution in our sample did not entirely meet the planned proportional stratification, all of the Portuguese regions were represented. Furthermore, we observed a relative homogeneity between regions regarding the rate of adherence to smoking ban. This finding suggests a relative independence of the region regarding the primary outcome of interest, minimizing the consequence of a lack of proportional representativeness of the regions.

Adherence to legislation and its policies were objectively measured by observing the venues *in loco*, increasing the robustness of the conclusions regarding this result.

There was no formal process to assess the accuracy and precision of the indicators of noncompliance. These indicators were defined by the authors, tailored to the specificities of the national smoking control policies. This approach conferred an appropriate empirical validity to this research when assessing the level of noncompliance to Portuguese legislation within this sector. However, it should be noted that the indicators used for each policy were obviously different, so one should be cautious when comparing noncompliance between policies.

To minimise information bias the same observer was allocated to the same locality. Moreover, all efforts were made by the observers not to make their presence known. This way, social desirability bias and potential sampling bias associated with the non-response was minimised. However, in spite of the use of standardised procedures we cannot ignore the potential for inter-observer variability.

Airborne and biological markers are also important to evaluate the effectiveness of smoke-free legislation. This data was also collected during our research and will be subject to analysis and publication in the near future.

## Conclusions

Globally, our results show a high acceptance to the smoking ban policy in Portugal in the LH sector, reflected by the large proportion of venues that adhered to that policy three years after it came into force. However, one quarter of the venues is totally or partially permissive towards smoking, with the discos/pubs/bars considerably contributing to this situation. We also conclude that places that adopt smoking permission policies are less compliant with the legislation.

In 2004 Portugal ratified the WHO Framework Convention on Tobacco Control (FCTC) that calls for parties to provide universal protection with no justification to exemptions on the basis of health or law arguments. Exceptions in the Portuguese smoke-free law clearly violate this convention. The Portuguese case reinforces the need to implement total ban policies worldwide, without partial restrictions, covering all enclosed public places. This is the only effective way to reduce the burden of disease related to SHS exposure by the workers and customers of the LH sector.

## Supporting Information

Table S1 **Indicators of noncompliance regarding each smoke-free policy.**
(ZIP)

Dataset S1 **Study Dataset.**
(ZIP)
