# Supplementary material for: Patterns of Adherence to and Compliance with the Portuguese Smoke-Free Law in the Leisure-Hospitality Sector
Source: PLoS One. 2014 Jul 18;9(7):e102421. doi: 10.1371/journal.pone.0102421 (PMC4103806; doi:10.1371/journal.pone.0102421)
Supplement: Table S1 — Indicators of noncompliance regarding each smoke-free policy. (ZIP) [file pone.0102421.s001.zip › Table S1.doc]

**Table S1** Indicators of noncompliance regarding each smoke-free policy

| **Signage visibility (overall)** | **n (%)** |
| --- | --- |
| From outdoors (n=1392): |  |
| Red display only | 879 (63.2) |
| Blue display only | 196 (14.1) |
| Red and blue displays | 110 (7.9) |
| Inexistent/Not visible | 207 (14.9) |
| From indoors (n=1412): |  |
| Red display only | 886 (62.7) |
| Blue display only | 178 (12.6) |
| Red and blue displays | 107 (7.6) |
| Inexistent/Not visible | 241 (17.1) |
| **Smoking ban venues – red display** | |
| Display with adequate dimension (n=885; missing=16): |  |
| No | 61 (6.9) |
| Yes | 824 (93.1) |
| Display with reference to smoke free law (n=877; missing=24): |  |
| No | 21 (2.4) |
| Yes | 856 (97.6) |
| Display with reference to the penalty for violating the smoking ban (n=870; missing=31): |  |
| No | 32 (3.7) |
| Yes | 838 (96.3) |
| Evidence of tobacco consumption: |  |
| People smoking | 25 (2.4) |
| Ashtrays | 35 (3.4) |
| Tobacco smell | 58 (5.6)* |
| Cigarette butt | 22 (2.2)* |
| **Venues with DSA – blue display** | |
| Smoking areas identified with blue display (n=104; missing=2): |  |
| No | 6 (5.8) |
| Yes | 98 (94.2) |
| Blue display with adequate dimension (n=100; missing=3): |  |
| No | 11 (11.0) |
| Yes | 89 (89.0) |
| Label with reference to smoke-free law (n=99; missing=4): |  |
| No | 3 (3.0) |
| Yes | 96 (97.0) |
| Physical separation between smoking and non-smoking areas (n=100; missing=6): | |
| No | 20 (20.0) |
| Yes | 80 (80.0) |
| Non-smoking areas (n=117): |  |
| People smoking | 16 (13.7)* |
| Environmental smoke | 20 (17.1) |
| Tobacco smell | 28 (23.9)* |
| Other evidence of smoking | 2 (1.7) |
| **Full smoking permission venues** | |
| Blue displays with adequate dimension (n=185): |  |
| No | 8 (4.3) |
| Yes | 177 (95.7) |
| Blue displays with reference to smoke-free law (n=185): |  |
| No | 4 (2.2) |
| Yes | 181 (97.8) |
| Existence of ventilation system (n=185) |  |
| No | 5 (2.7) |
| Yes | 144 (77.8) |
| Non perceptible/not visible | 36 (19.5) |
| Working ventilation (n=172; missing=9) |  |
| No | 24 (14.0) |
| Yes | 82 (47.7) |
| Non perceptible | 66 (38.4) |

*The comparison between the indicated signs of tobacco use between smoking ban and DSA venues was statistically significant (Chi-Squared test; p<0.001); DSA – designated smoking areas.
